# Physiological responses and tolerance of *Idesia polycarpa* seedlings under simulated rhizosphere acidification

**DOI:** 10.3389/fpls.2026.1967073

**Published:** 2026-10-26

**Authors:** Zuwei Hu, Wenwen Zhong, Yanan Ma, Can Cao, Yulan Bei, Jiajia Hou, Yaohui Liu, Yanmei Wang, Li Dai, Qifei Cai, Xiaodong Geng, Juan Wang, Yongyu Ren, Fangming Liu, Zhen Liu, Hongzhu Zou, Shaoyu Chen, Tailin Zhong, Zhi Li

**Affiliations:** 1 College of Forestry, Henan Agricultural University, Zhengzhou, China; 2 College of Urban Construction, Zhejiang Shuren University, Shaoxing, China; 3 Henan Province Engineering Technology Research Center for Idesia, Zhengzhou, China; 4 Key Laboratory for Idesia polycarpa, National Forestry and Grassland Administration, Guiyang, China

**Keywords:** acidification stress, antioxidant defense, Idesia polycarpa, membrane lipid peroxidation, osmotic adjustment, photosynthetic pigments

## Abstract

**Introduction:**

Soil acidification poses a growing threat to forest seedling establishment, yet the physiological responses and tolerance limits of many multipurpose woody species under increasing acidity remain poorly understood. *Idesia polycarpa* Maxim. is an emerging woody oil tree with high ecological and economic value, but its physiological responses to root-zone acidification are largely unknown.

**Methods:**

We subjected one-year-old *I. polycarpa* seedlings to 50 d of root-zone irrigation with acidified solutions at pH 5.5, 4.5, and 3.5, with deionized water as the control, and monitored visible injury, photosynthetic pigments, malondialdehyde (MDA), osmotic adjustment substances, and the activities of peroxidase (POD) and superoxide dismutase (SOD).

**Results:**

Visible injury increased with both acidity and exposure duration, and the cumulative injury index reached 85.00% under pH 3.5, 40.00% under pH 4.5, and 16.67% under pH 5.5 after 50 d, while CK remained at 0%. Under pH 3.5, photosynthetic pigment contents showed pronounced temporal fluctuations, with marked reductions at 20 and 50 d and partial recovery during the intermediate stages. MDA content also exhibited a non-monotonic response, with significantly higher values under pH 3.5 at several middle and late sampling times. Soluble sugar, proline, and soluble protein showed stage- and treatment-dependent changes rather than continuous accumulation or decline. POD activity under pH 3.5 reached approximately 3.55-fold the CK value at 10 d and subsequently declined, whereas SOD activity fluctuated markedly among treatments and sampling times. Correlation analysis revealed coordinated variation among photosynthetic pigments, MDA, osmotic adjustment substances, and the two measured antioxidant enzymes, but these associations do not establish causal relationships.

**Discussion:**

Overall, seedlings under pH 5.5 and pH 4.5 showed partial physiological adjustment, whereas pH 3.5 was associated with substantially greater visible injury and physiological disturbance. The contrasting responses between the pH 4.5 and pH 3.5 irrigation treatments suggest a potential physiological tolerance boundary within this tested acidity interval under the present controlled conditions, rather than a statistically defined biological breakpoint.

## 1 Introduction

Against the backdrop of global environmental change and intensified human activities, soil acidification has become an important environmental issue affecting the structure, function, and productivity of forest ecosystems. Sulfur and nitrogen deposition derived from industrial activities, together with excessive nitrogen fertilizer input in agricultural systems, can continuously accelerate soil acidification processes (Zhang et al., 2022; Xie et al., 2024). In China, forest soil acidification and acid deposition are particularly pronounced in southern and humid subtropical forest regions, where they may trigger a series of ecological effects, including declines in soil pH, losses of base cations, and alterations in forest ecological processes (Yu et al., 2020; Wang et al., 2023; Xie et al., 2024).

Soil acidification can suppress the growth and establishment of woody plants through multiple physiological and ecological pathways. Acid deposition and persistent acidification may accelerate the leaching of base cations such as Ca, Mg, and K, thereby reducing their availability to plants (Yu et al., 2020; Wang et al., 2023). Meanwhile, increasing acidity can enhance the mobilization of metal ions, particularly aluminum, which may cause nutrient imbalance and root toxicity (Rahman et al., 2018; Wang et al., 2023). Previous studies have shown that toxic Al³^+^ in acidic soils can rapidly inhibit root elongation and subsequently impair water and nutrient uptake by plants (Rahman et al., 2018). Over time, acidification stress may further disrupt tree growth by inducing root damage, reducing membrane stability, and promoting oxidative stress accumulation (Rahman et al., 2018; Wang et al., 2023). However, the relative contributions of proton stress, Al mobilization, and acidification-induced nutrient imbalance depend strongly on soil properties and cannot be inferred from solution pH alone.

*I. polycarpa* Maxim. is a woody tree species with considerable oil-producing, ecological, and economic value. Its fruits and fruit oil are rich in polyunsaturated fatty acids and various bioactive compounds, indicating substantial potential for further development and utilization (Zhang et al., 2023a; Zuo et al., 2024). Existing studies have suggested that *I*. *polycarpa* has promising potential for afforestation, ecological utilization, and cultivation in certain mountainous areas and marginal lands, which is consistent with the demand for developing non-grain woody oil resources and improving oil and bioenergy security in China (Li et al., 2023; Wu et al., 2024).

However, current research on *I. polycarpa* has focused mainly on fruit composition and oil quality, oil biosynthesis and genomics, germplasm resources, genetic improvement, fertilization, and cultivation management (Guo et al., 2023; Zhang et al., 2023a; Luo et al., 2024; Zuo et al., 2024; Miao et al., 2026). In contrast, relatively little attention has been paid to the physiological responses of *I. polycarpa* seedlings to acidification stress, particularly the changes in photosynthetic pigments, membrane lipid peroxidation, osmotic adjustment substances, and antioxidant enzyme activities under increasing acidity. It remains unclear how *I. polycarpa* seedlings respond physiologically to increasing root-zone acidification and whether a transition from partial physiological adjustment to pronounced stress injury can be identified under controlled conditions.

Therefore, in this study, one-year-old *I. polycarpa* seedlings were subjected to root-zone irrigation with acidified solutions at different pH levels to impose controlled root-zone acidification stress. Visible injury symptoms, photosynthetic pigment contents, membrane lipid peroxidation, osmotic adjustment substances, and antioxidant enzyme activities were systematically measured over a 50-day treatment period. The objectives of this study were to: (1) characterize the dynamic physiological responses of *I. polycarpa* seedlings under different levels of controlled root-zone acidification; (2) examine the coordinated changes and statistical associations among photosynthetic pigments, membrane lipid peroxidation, osmotic adjustment substances, and the measured antioxidant enzyme activities; and (3) evaluate whether the observed physiological responses indicate a potential tolerance boundary under controlled acidification conditions. The findings provide physiological evidence for evaluating the acidification tolerance of *I. polycarpa* and may inform its cultivation and preliminary suitability assessment in acidic or acidification-prone sites.

## 2 Materials and methods

### 2.1 Experimental site and plant materials

The experiment was conducted at the Forestry Experimental Station of Henan Agricultural University, Zhengzhou, China. The study site has a warm temperate monsoon climate, with an annual mean temperature of approximately 14.2 °C, annual precipitation of about 650.1 mm, approximately 2400 h of sunshine, and a frost-free period of about 215 d.

One-year-old seedlings of *I. polycarpa* Maxim. ‘Yitong No. 1’ were used as experimental materials. Seeds were sown in March 2024 and transplanted into pots in June 2024. The nursery substrate consisted of vermiculite and peat at a volume ratio of 1:1. The substrate had an available nitrogen content of approximately 1.32 g kg^−^¹, available phosphorus content of 0.022 g kg^−^¹, available potassium content of 1.29 g kg^−^¹, pH of approximately 6.45, and maximum field water-holding capacity of about 65%.

The pots used in the experiment had an outer diameter of 29.6 cm, an inner diameter of 25.4 cm, a height of 19.7 cm, and a bottom diameter of 17.8 cm. In May 2025, seedlings with uniform growth, no visible pests or diseases, and similar vigor were selected for the experiment. Before treatment, the selected seedlings had an average height of approximately 55.8 cm and an average basal stem diameter of approximately 1.1 cm.

### 2.2 Experimental design and simulated acidification treatment

After transplanting, all seedlings were cultivated under the same nursery conditions. In May 2025, uniformly growing seedlings were selected and acclimated for 30 d before the acidification treatment. During the acclimation period, all seedlings were managed under consistent water and light conditions. Before treatment, the seedlings were irrigated with deionized water for 3 d to minimize differences in initial water status.

A total of 60 seedlings were randomly assigned to four treatments, with 15 seedlings per treatment. The treatments included a neutral control (CK) and three acidification treatments at pH 5.5, pH 4.5, and pH 3.5. The neutral control was irrigated with deionized water, whereas the acidification solutions were prepared from 98% H_2_SO_4_ and deionized water and adjusted to the target pH values before application. The pH of each solution was adjusted to the target value from the beginning of the treatment and verified using a calibrated pH meter before each application. Hereafter, the treatment labels pH 5.5, pH 4.5, and pH 3.5 refer to the pH of the applied irrigation solutions rather than the actual substrate pH.

In this experiment, acidification stress in the root zone was simulated by applying acidified solutions to the rhizosphere of potted seedlings. The treatment lasted for 50 d, and the corresponding treatment solution was applied once every 5 d at a volume of 250 mL per pot. During the treatment period, all seedlings were maintained under the same environmental and management conditions. To avoid cross-contamination among treatments, pots from different treatments were kept separately, and irrigation was conducted carefully to prevent solution overflow between pots.

Three additional homogeneous seedlings were sampled before treatment as the 0-d baseline group. These seedlings were used only to represent the initial physiological status before acidification treatment and were not included in the 60 seedlings assigned to the four treatment groups.

### 2.3 Sample collection

Leaf samples were collected at 0, 10, 20, 30, 40, and 50 d after the start of acidification treatment. The 0-d samples were collected from three additional homogeneous seedlings before treatment and were used as independent biological replicates representing the initial baseline. For the neutral control and acidification treatments, samples were collected at 10, 20, 30, 40, and 50 d.

At each post-treatment sampling time, three previously unsampled seedlings were randomly selected from each treatment and destructively harvested. Each potted seedling was considered an independent experimental unit and biological replicate (n = 3 per treatment at each sampling time). Because different seedlings were sampled at successive time points, no individual seedling contributed physiological or biochemical measurements to more than one post-treatment sampling time. The third to fifth fully expanded healthy leaves from the top of each seedling were collected for physiological and biochemical analyses. After collection, leaf samples were immediately frozen in liquid nitrogen and stored at −80 °C until analysis.

Each individual seedling was considered an independent biological replicate. For each biological replicate, leaves collected from the same seedling were pooled and homogenized to obtain one biological sample. The resulting extract was measured in triplicate as technical replicates for each physiological or biochemical assay, and the mean of the three technical measurements was used as the value of that biological replicate for subsequent statistical analysis.

### 2.4 Determination of physiological and biochemical parameters

#### 2.4.1 Grading of seedling injury symptoms and calculation of the cumulative injury index

Visible injury symptoms of *I. polycarpa* seedlings were assessed before treatment (0 d) and at 10-d intervals during the 50-d treatment period. Before destructive sampling at each observation time, the injury grade of each remaining seedling was recorded according to the severity of leaf chlorosis, wilting, necrosis, marginal scorch, defoliation, and plant death. Injury severity was classified into five grades according to the criteria shown in **Table 1**. Because three seedlings per treatment were destructively harvested at each post-treatment sampling time, seedlings harvested at an earlier sampling time were no longer available for subsequent visual assessment. For these harvested seedlings, the last injury grade recorded immediately before harvest was retained in subsequent cumulative calculations, whereas the injury grades of the remaining seedlings were updated at each subsequent observation. Therefore, the cumulative injury index at each sampling time was calculated using the most recent available injury grade for each of the original 15 seedlings in a treatment cohort.

**Table 1:** Grading criteria for injury in *I. polycarpa* seedlings under simulated acidification stress.

Grade	Assigned value	Injury symptoms
I	0	Plants grew normally, and the leaves were green and fully expanded, with no visible chlorosis or necrotic spots.
II	1	A very small number of lower old leaves showed slight marginal chlorosis or yellowing, with the damaged leaf area accounting for<25%
III	2	Leaves exhibited obvious yellowing, and the leaf margins began to show desiccation, curling, or water-soaked necrotic lesions, with 25% ≤ damaged leaf area< 50%.
IV	3	Most leaves showed severe yellowing and wilting, with an expanded area of necrosis and desiccation; some leaves had fallen off, with 50% ≤ damaged leaf area< 75%.
V	4	Most leaves were desiccated, necrotic, and extensively abscised, with damaged leaf area ≥75%; the shoot apex was withered, or even the whole plant had died.

The cumulative injury index was calculated using the following formula:


DI=(∑(n∗v))/(N∗V)∗100

The variables in the formula are defined as follows: DI represents the cumulative injury index; n is the number of seedlings assigned to a given injury grade based on the most recent available observation; v is the numerical value assigned to the corresponding injury grade; N is the initial number of seedlings in each treatment cohort (N = 15); and V is the numerical value of the highest injury grade (V = 4).

#### 2.4.2 Determination of photosynthetic pigment contents

Photosynthetic pigment contents were determined using the 95% ethanol extraction method (Lichtenthaler, 1987). Fresh leaf samples were cut into small pieces and extracted with 95% ethanol in the dark until the tissues were completely decolorized. The absorbance of the extract was measured at 665, 649, and 470 nm using a spectrophotometer. The contents of chlorophyll a, chlorophyll b, total chlorophyll, and carotenoids were calculated according to standard equations and expressed on a fresh weight basis.

#### 2.4.3 Determination of membrane lipid peroxidation

The degree of membrane lipid peroxidation was evaluated by determining malondialdehyde content using the thiobarbituric acid method (Heath and Packer, 1968). Fresh leaf samples were homogenized in trichloroacetic acid solution and centrifuged. The supernatant was mixed with thiobarbituric acid reagent and heated in a boiling water bath. After cooling and centrifugation, the absorbance of the reaction mixture was measured spectrophotometrically. Malondialdehyde content was calculated according to standard equations and expressed on a fresh weight basis.

#### 2.4.4 Determination of osmotic adjustment substances

Soluble sugar content was determined using the anthrone colorimetric method (Yemm and Willis, 1954). Fresh leaf samples were extracted, reacted with anthrone reagent under heating, and the absorbance was measured using a spectrophotometer. Soluble sugar content was calculated from a standard curve prepared with glucose and expressed on a fresh weight basis.

Proline content was determined using the acid ninhydrin method (Bates et al., 1973). Fresh leaf samples were extracted with sulfosalicylic acid, and the extract was reacted with acid ninhydrin reagent. After heating and extraction with toluene, the absorbance of the colored phase was measured spectrophotometrically. Proline content was calculated using a standard curve and expressed on a fresh weight basis.

Soluble protein content was determined using the Coomassie Brilliant Blue G-250 method (Bradford, 1976). The absorbance was measured spectrophotometrically, and soluble protein content was calculated using a standard curve prepared with bovine serum albumin.

#### 2.4.5 Determination of antioxidant enzyme activities

Superoxide dismutase activity was determined using the nitroblue tetrazolium photoreduction method (Beauchamp and Fridovich, 1971). Fresh leaf samples were homogenized in phosphate buffer and centrifuged, and the supernatant was used for enzyme activity determination. One unit of superoxide dismutase activity was defined as the amount of enzyme required to inhibit 50% of nitroblue tetrazolium reduction under the assay conditions.

Peroxidase activity was determined using the guaiacol colorimetric method (Chance and Maehly, 1955). The reaction mixture contained enzyme extract, phosphate buffer, guaiacol, and hydrogen peroxide. The change in absorbance caused by guaiacol oxidation was recorded spectrophotometrically, and peroxidase activity was calculated according to the change in absorbance per unit time.

#### 2.4.6 Determination of post-treatment substrate pH

At the end of the 50-d treatment period, a composite substrate sample was prepared for each treatment by combining substrate collected from three pots within the same treatment. Substrate pH was determined using a substrate-to-deionized-water ratio of 1:2.5 (w/v). The suspension was thoroughly mixed, and pH was measured using a KN-P901 pH meter. Each composite sample was measured three times as technical replicates, and the mean of the three readings was used as the post-treatment substrate pH value for that treatment. Because only one composite substrate sample was obtained for each treatment, these pH data were used descriptively and were not subjected to inferential statistical analysis.

### 2.5 Data processing and statistical analysis

All data were organized using Microsoft Excel and analyzed using SPSS 26.0. Data are presented as mean ± standard deviation (SD). For physiological and biochemical parameters, each potted seedling was treated as an independent experimental unit and biological replicate. The mean of three technical measurements was first calculated for each biological replicate, and statistical analyses were then performed using the three independent biological replicates per treatment at each sampling time (n = 3). All inferential statistical analyses were conducted using individual biological-replicate values rather than treatment-averaged values. Because destructive sampling was used, different seedlings were analyzed at 10, 20, 30, 40, and 50 d, and no individual seedling was measured repeatedly across sampling times. Therefore, the post-treatment observations were treated as independent rather than repeated measurements. Data normality and homogeneity of variance were tested before analysis of variance. Post-treatment data collected at 10, 20, 30, 40, and 50 d were analyzed using two-way ANOVA, with acidity treatment and sampling time as fixed factors, including their interaction. When significant differences were detected, Duncan’s multiple range test was used for *post hoc* comparisons at P< 0.05. The 0-d samples represented a common pretreatment baseline and were not included in the factorial ANOVA. Pearson correlation analysis was performed to assess relationships among photosynthetic pigment contents, membrane lipid peroxidation, osmotic adjustment substances, and the measured antioxidant enzyme activities. Figures were generated using Origin 2025b.

## 3 Results

### 3.1 Post-treatment substrate pH, visible injury symptoms, and cumulative injury index under simulated acidification stress

At the end of the 50-d treatment period, the pH of the composite substrate samples was 6.86 in CK, 5.63 in the pH 5.5 treatment, 5.33 in the pH 4.5 treatment, and 4.99 in the pH 3.5 treatment. Endpoint substrate pH decreased progressively with increasing acidity of the applied solutions. In the acidification treatments, the endpoint substrate pH values remained higher than the nominal pH values of the corresponding irrigation solutions, a pattern consistent with buffering by the growth substrate. Because one composite substrate sample was analyzed per treatment, these endpoint pH values are presented descriptively.

Visible injury symptoms and the cumulative injury index showed progressively greater changes with increasing acidity and treatment duration (**Table 2**). At 0 d, seedlings in all treatments were healthy, with green and fully expanded leaves and no visible injury symptoms. At 10 d, seedlings in CK and pH 5.5 showed little visible injury, whereas slight marginal chlorosis of older leaves was observed under pH 3.5. The cumulative injury index was 8.33% under pH 3.5, compared with 3.33% under pH 4.5, 1.67% under pH 5.5, and 0% in CK.

**Table 2:** Cumulative injury index of *I. polycarpa* seedlings under simulated acidification stress.

Treatment duration (d)	Treatment	Cumulative injury index (%)
0	CK,5.5,4.5,3.5	0.00
10	CK	0.00
	5.5	1.67
	4.5	3.33
	3.5	8.33
20	CK	0.00
	5.5	6.67
	4.5	13.33
	3.5	26.67
30	CK	0.00
	5.5	10.00
	4.5	23.33
	3.5	46.67
40	CK	0.00
	5.5	15.00
	4.5	36.67
	3.5	73.33
50	CK	0.00
	5.5	16.67
	4.5	40.00
	3.5	85.00

With prolonged treatment, visible chlorosis, yellowing, wilting, and leaf desiccation became increasingly apparent in the stronger acidification treatments. At 20 and 30 d, the cumulative injury index under pH 3.5 increased to 26.67% and 46.67%, respectively, whereas the corresponding values under pH 4.5 were 13.33% and 23.33%. By 40 d, severe symptoms, including extensive leaf desiccation, wilting, and abscission, were observed under pH 3.5, and the cumulative injury index reached 73.33%, compared with 36.67% under pH 4.5 and 15.00% under pH 5.5.

At 50 d, the strongest visible injury occurred under pH 3.5, including branch desiccation and near-death symptoms. The cumulative injury index reached 85.00% under pH 3.5, whereas values of 40.00% and 16.67% were recorded under pH 4.5 and pH 5.5, respectively; CK remained at 0%. Overall, the cumulative injury index showed a clear increase with treatment duration and increasing acidity, with substantially greater cohort-level injury under pH 3.5 than under the milder acidification treatments.

### 3.2 Changes in photosynthetic pigment contents under simulated acidification stress

The contents of chlorophyll a (Chl a), chlorophyll b (Chl b), total chlorophyll (Total Chl), and carotenoids (Car) in *I. polycarpa* leaves showed clear acidity- and time-dependent changes under simulated acidification stress (**Figure 1**).

**Figure 1:**
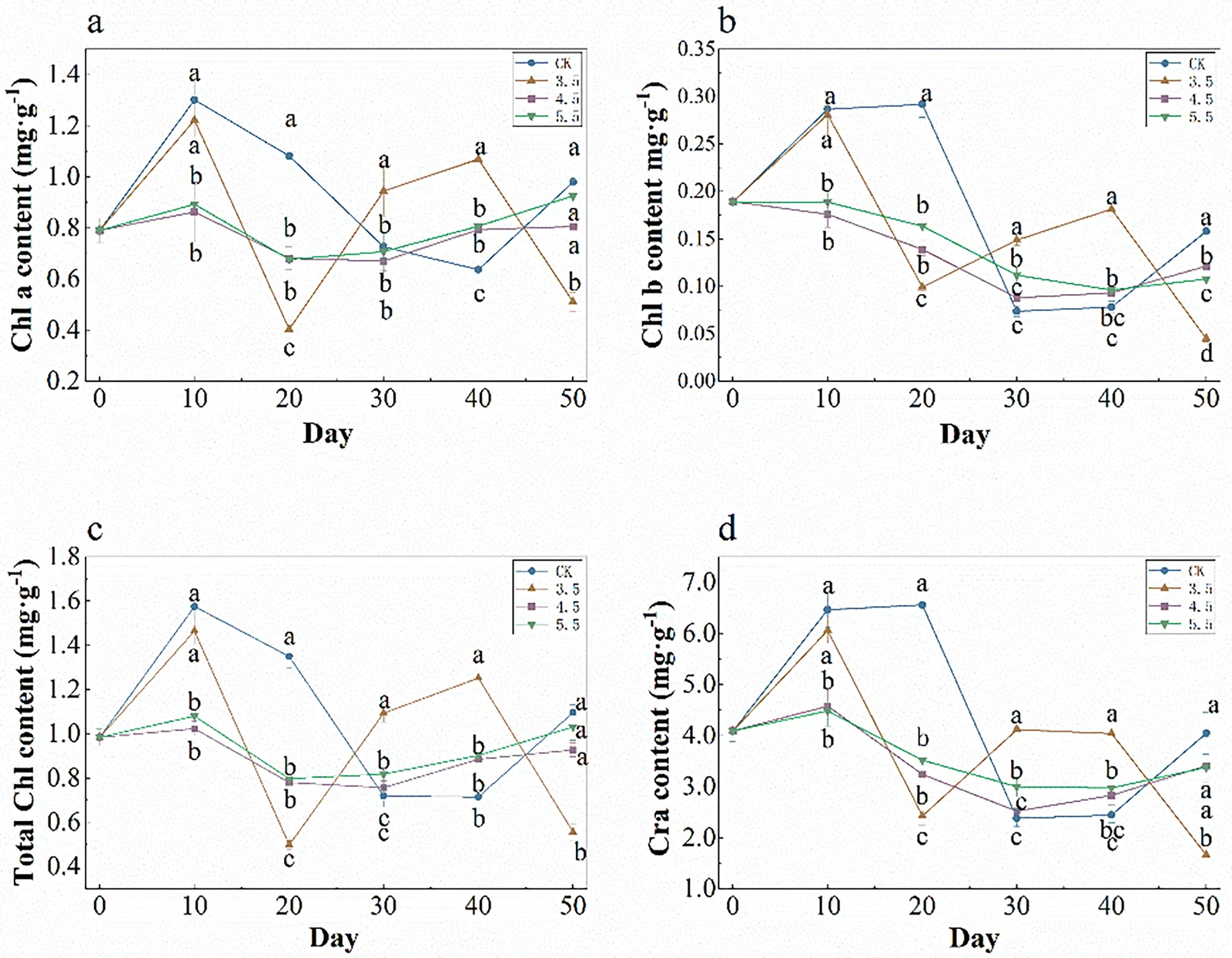
Dynamic changes in photosynthetic pigment contents in leaves of *I. polycarpa* seedlings under simulated acidification stress. **(a)** Chlorophyll a content; **(b)** chlorophyll b content; **(c)** total chlorophyll content; **(d)** carotenoid content. Data are presented as mean ± SD (n = 3 independent biological replicates per treatment at each sampling time). Different lowercase letters indicate significant differences among treatments at the same sampling time according to Duncan’s multiple range test following two-way ANOVA (P< 0.05).

As shown in **Figure 1a**, Chl a content exhibited distinct temporal fluctuations among the treatments. Relative to the 0-d baseline, the mean Chl a content increased at 10 d in CK and the pH 3.5 treatment, whereas only minor changes were observed under pH 4.5 and pH 5.5. At 20 d, Chl a content under pH 3.5 decreased sharply and was approximately 62.7% lower than that in CK, and was also significantly lower than that in the pH 4.5 and pH 5.5 treatments (P< 0.05). Subsequently, Chl a content under pH 3.5 increased markedly at 30 and 40 d and was significantly higher than that in the other treatments at both sampling times (P< 0.05). At 50 d, however, Chl a content under pH 3.5 declined again, reaching a value approximately 47.9% lower than that in CK, and was significantly lower than that in the other treatments (P< 0.05). Thus, the pH 3.5 treatment showed a pronounced fluctuating response rather than a sustained decline during the middle and late stages of treatment.

As shown in **Figure 1b**, Chl b content exhibited clear temporal changes under the different treatments. Under pH 3.5, Chl b content decreased markedly at 20 d and reached its lowest level at 50 d. At 50 d, Chl b content under pH 3.5 was significantly lower than that in CK, pH 4.5, and pH 5.5 (P< 0.05). Chl b content under pH 4.5 and pH 5.5 also declined during the treatment period, indicating that changes in Chl b were not restricted to the most severe acidification treatment. Overall, the strongest late-stage reduction in Chl b was observed under pH 3.5.

As shown in **Figure 1c**, Total Chl content exhibited pronounced temporal fluctuations among the treatments. At 10 d, relatively high Total Chl contents were observed in CK and the pH 3.5 treatment, whereas lower values were observed under pH 4.5 and pH 5.5. From 10 to 20 d, Total Chl content decreased in all treatments, with the most pronounced reduction occurring under pH 3.5. At 20 d, Total Chl content under pH 3.5 was significantly lower than that in CK, pH 4.5, and pH 5.5 (P< 0.05). Subsequently, Total Chl content under pH 3.5 increased again at 30 and 40 d, but declined markedly by 50 d. Overall, the pH 3.5 treatment showed a fluctuating rather than continuously decreasing pattern in Total Chl content.

As shown in **Figure 1d**, Car content exhibited treatment-specific temporal fluctuations. At 10 d, Car content increased markedly in CK and the pH 3.5 treatment, whereas only minor changes were observed under pH 4.5 and pH 5.5 relative to the 0-d baseline. At 20 d, Car content under pH 3.5 decreased sharply and was significantly lower than that in CK (P< 0.05). Thereafter, Car content under pH 3.5 increased again at 30 and 40 d, followed by another marked decline at 50 d. These results indicate that Car content under severe acidification showed a pronounced fluctuating response rather than a simple transient increase followed by a continuous decline.

Overall, photosynthetic pigment contents exhibited pronounced treatment- and time-dependent fluctuations under simulated acidification stress. The pH 3.5 treatment showed the greatest temporal variability, with marked reductions in pigment contents at specific sampling times, particularly at 20 and 50 d, together with partial recovery of several pigments during the intermediate stages. These results indicate that severe acidification induced an unstable rather than continuously declining photosynthetic pigment response in *I. polycarpa* seedlings.

### 3.3 Changes in membrane lipid peroxidation and osmotic adjustment substances

MDA content and osmotic adjustment substances in *I. polycarpa* leaves showed distinct changes in response to simulated acidification stress (**Figure 2**).

**Figure 2:**
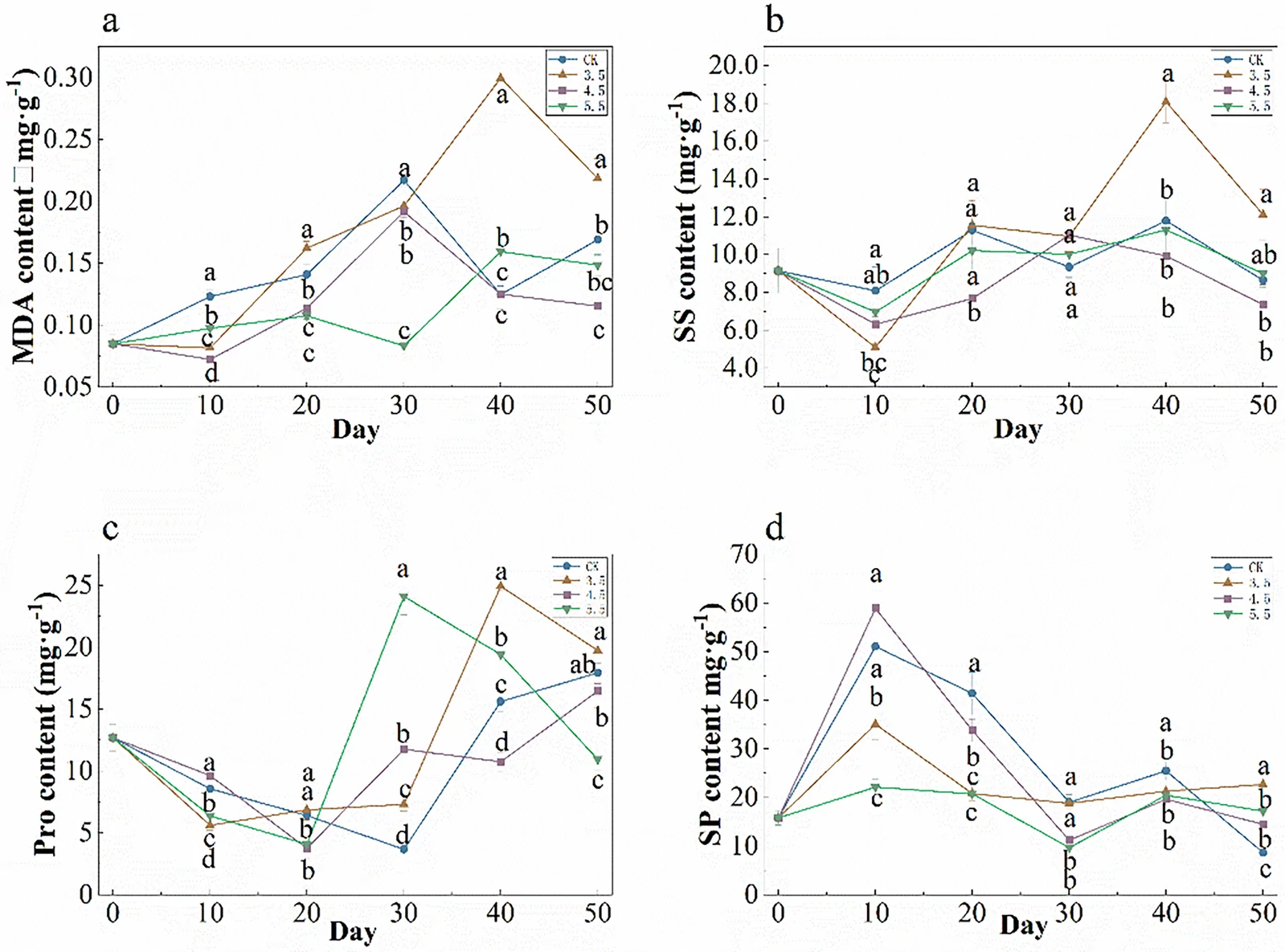
Dynamic changes in membrane lipid peroxidation and osmotic adjustment substances in leaves of *I. polycarpa* seedlings under simulated acidification stress. **(a)** MDA content; **(b)** soluble sugar content; **(c)** proline content; **(d)** soluble protein content. Data are presented as mean ± SD (n = 3 independent biological replicates per treatment at each sampling time). Different lowercase letters indicate significant differences among treatments at the same sampling time according to Duncan’s multiple range test following two-way ANOVA (P< 0.05).

As shown in **Figure 2a**, MDA content exhibited a non-monotonic response to simulated acidification stress. At 10 d, MDA content was highest in CK, whereas lower values were observed in the acidification treatments. At 20 d, MDA content under pH 3.5 increased and was significantly higher than that in CK, pH 4.5, and pH 5.5 (P< 0.05). At 30 d, CK showed the highest MDA content, whereas the pH 3.5 and pH 4.5 treatments showed intermediate values and pH 5.5 showed the lowest value. At 40 d, MDA content under pH 3.5 increased sharply and was significantly higher than that in all other treatments (P< 0.05). By 50 d, MDA content under pH 3.5 had declined from its 40-d peak but remained significantly higher than that in CK, pH 4.5, and pH 5.5 (P< 0.05). Overall, severe acidification induced a delayed and fluctuating increase in membrane lipid peroxidation rather than a continuous increase throughout the treatment period.

As shown in **Figure 2b**, soluble sugar content exhibited a fluctuating temporal response among the treatments. At 10 d, soluble sugar content decreased relative to the 0-d baseline in all treatments, followed by an increase at 20 d. At 30 d, the four treatments showed comparable soluble sugar levels, with no clear separation among treatments. At 40 d, soluble sugar content under pH 3.5 was approximately 53.2% higher than that in CK and reached the highest mean level among the treatments. From 40 to 50 d, soluble sugar content declined in all treatments, although the value under pH 3.5 remained relatively high at the final sampling time. These results indicate that soluble sugar accumulation was stage-specific rather than continuously increasing with treatment duration.

As shown in **Figure 2c**, proline content exhibited a highly variable and treatment-specific temporal response. Relative to the 0-d baseline, proline content decreased in all treatments during the early stage, with generally lower values observed at 10 and 20 d. At 30 d, proline content increased markedly under pH 5.5, reaching approximately 6.55-fold the CK value, and represented the highest mean level among the treatments, whereas the pH 3.5 treatment remained relatively low. At 40 d, proline content increased substantially under pH 3.5, reaching a level approximately 59.5% higher than that in CK and the highest mean value among the treatments. By 50 d, proline content remained relatively high under pH 3.5, CK, and pH 4.5, whereas it decreased under pH 5.5. Overall, proline accumulation showed a pronounced stage- and treatment-dependent pattern rather than a progressive increase with treatment duration.

As shown in **Figure 2d**, soluble protein content exhibited pronounced temporal and treatment-dependent fluctuations. At 10 d, soluble protein content increased markedly in all treatments relative to the 0-d baseline, with the highest mean value observed under pH 4.5, followed by CK. Thereafter, soluble protein content generally declined, and relatively low levels were observed across treatments at 30 d. A partial recovery occurred at 40 d. By 50 d, soluble protein content declined further in CK, whereas comparatively higher values were maintained in the acidification treatments, particularly under pH 3.5. Overall, soluble protein content showed an early increase followed by a decline and subsequent treatment-dependent fluctuations rather than a sustained late-stage decrease specifically under severe acidification.

Overall, MDA, soluble sugar, proline, and soluble protein exhibited distinct stage- and treatment-dependent responses to simulated acidification stress. Severe acidification at pH 3.5 produced pronounced fluctuations in these physiological parameters, including a marked increase in MDA during the later treatment stage and transient increases in soluble sugar and proline at specific sampling times. However, none of the osmotic adjustment parameters showed a simple or continuously increasing response with treatment duration. These results indicate that membrane lipid peroxidation and osmotic adjustment responses were highly dynamic during acidification treatment.

### 3.4 Changes in antioxidant enzyme activities under simulated acidification stress

POD and SOD activities in *I. polycarpa* leaves exhibited distinct temporal and treatment-dependent responses to simulated acidification stress (**Figure 3**).

**Figure 3:**
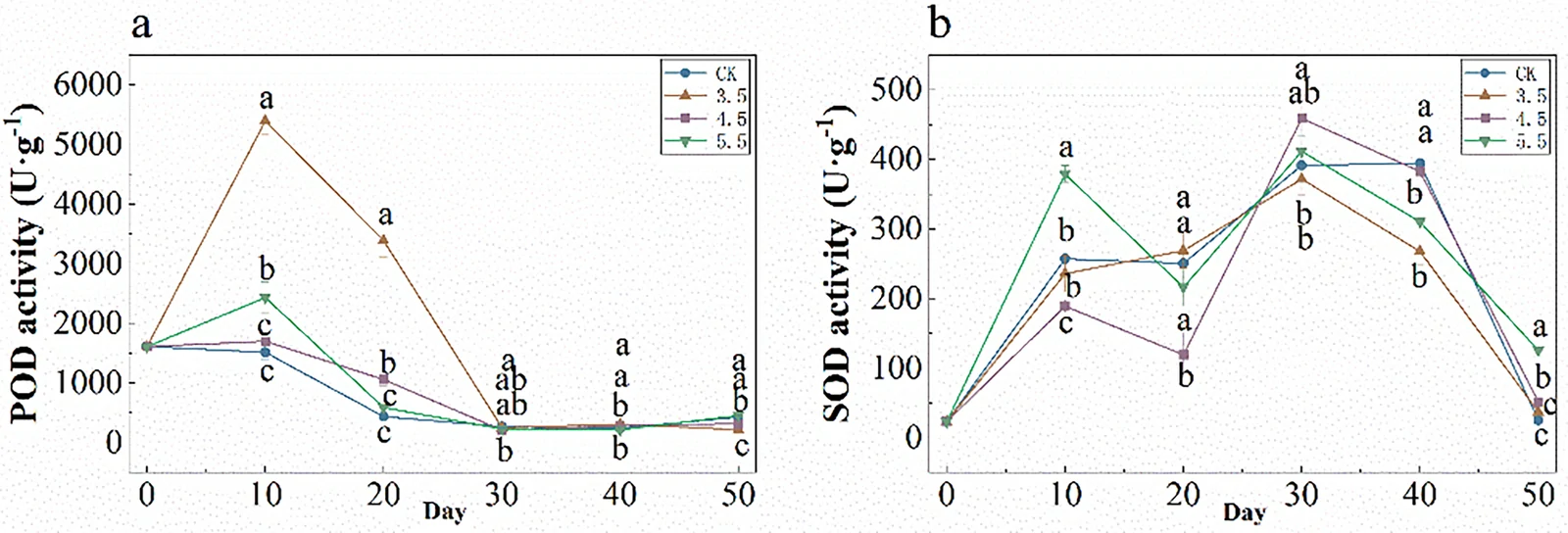
Dynamic changes in antioxidant enzyme activities in leaves of *I. polycarpa* seedlings under simulated acidification stress. **(a)** POD activity; **(b)** SOD activity. Data are presented as mean ± SD (n = 3 independent biological replicates per treatment at each sampling time). Different lowercase letters indicate significant differences among treatments at the same sampling time according to Duncan’s multiple range test following two-way ANOVA (P< 0.05).

As shown in **Figure 3a**, POD activity responded rapidly to acidification treatment during the early stage. At 10 d, POD activity under pH 3.5 reached approximately 3.55-fold the CK value and represented the highest mean activity among the treatments, whereas a more moderate increase was observed under pH 5.5. POD activity under pH 3.5 remained relatively high at 20 d, while lower values were observed in the other treatments. By 30 d, POD activity had declined markedly across all treatments and subsequently remained at relatively low levels through 40 and 50 d. Thus, the pH 3.5 treatment induced a pronounced but transient early POD response that was not sustained during prolonged exposure.

As shown in **Figure 3b**, SOD activity showed substantial temporal fluctuations in all treatments. At 10 d, the highest mean SOD activity was observed under pH 5.5, whereas the other treatments showed comparatively lower values. At 20 d, pH 3.5 showed the highest mean value, while pH 4.5 showed the lowest. SOD activity increased again at 30 d in all treatments, with the highest mean value occurring under pH 4.5. At 40 d, relatively high SOD activity was maintained in CK and the pH 4.5 treatment, whereas a lower value was observed under pH 3.5. By 50 d, SOD activity declined markedly in all treatments, although the pH 5.5 treatment retained the highest mean value. Overall, SOD activity exhibited a strongly stage-dependent response rather than a sustained increase under any single acidification treatment.

Overall, POD and SOD displayed distinct temporal response patterns under simulated acidification stress. POD showed a pronounced early activation, particularly under pH 3.5, followed by a substantial decline during prolonged treatment. In contrast, SOD activity fluctuated among treatments throughout the experimental period, with the treatment showing the highest activity varying among sampling times. These results indicate that the two measured antioxidant enzymes responded differently to acidification intensity and exposure duration.

### 3.5 Correlations among physiological and biochemical parameters

As shown in **Figure 4**, Pearson correlation analysis was used to examine the statistical associations among photosynthetic pigment contents, membrane lipid peroxidation, osmotic adjustment substances, and the measured antioxidant enzyme activities under simulated acidification stress.

**Figure 4:**
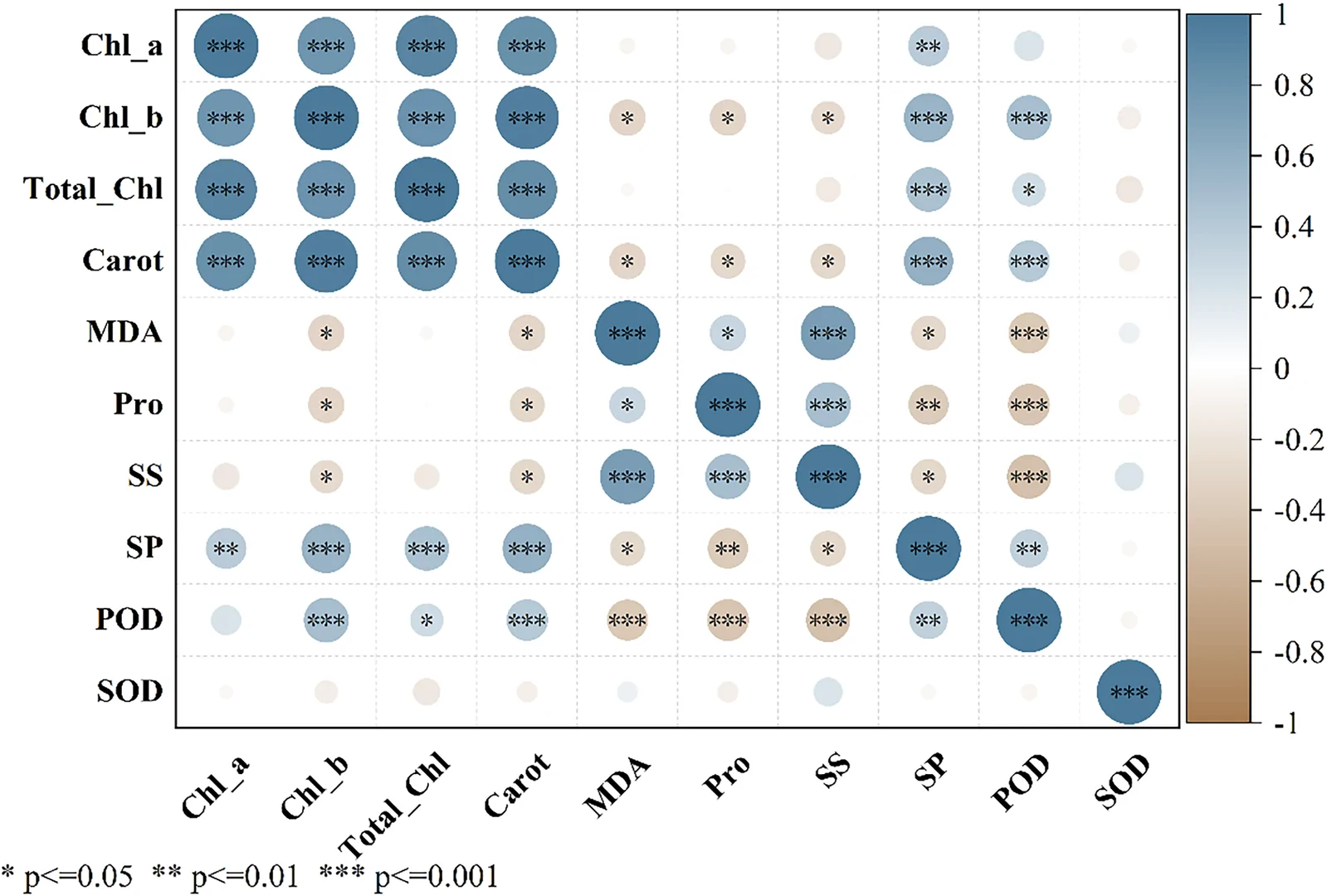
Pearson correlation analysis among physiological and biochemical parameters of *I. polycarpa* seedlings under simulated acidification stress. Chl a, chlorophyll a; Chl b, chlorophyll b; Total Chl, total chlorophyll; Car, carotenoids; MDA, malondialdehyde; SS, soluble sugar; Pro, proline; SP, soluble protein; SOD, superoxide dismutase; POD, peroxidase. Pearson correlation analysis was performed using individual biological-replicate values (n = 63) rather than treatment-averaged values. Statistical significance is indicated by *P< 0.05, **P< 0.01, and ***P< 0.001.

The photosynthetic pigment parameters were positively correlated with each other. Chl a, Chl b, Total Chl, and Car showed significant or highly significant positive correlations, reflecting coordinated variation among the photosynthetic pigments during the treatment period. Soluble protein content was also positively associated with photosynthetic pigment contents to varying degrees.

MDA content was negatively correlated with photosynthetic pigment contents, indicating an inverse statistical association between membrane lipid peroxidation and pigment levels during acidification treatment. In contrast, MDA content was positively correlated with soluble sugar and proline contents, showing that these osmotic adjustment variables tended to vary in parallel with MDA under the imposed treatments. These correlations describe coordinated changes among the measured parameters but do not establish a causal relationship between membrane lipid peroxidation and osmotic adjustment.

Among the two measured antioxidant enzymes, POD activity showed stronger statistical associations with other physiological parameters than SOD activity. POD activity was positively correlated with several photosynthetic pigment parameters and negatively correlated with MDA, soluble sugar, and proline contents. In comparison, SOD activity showed relatively weak or non-significant correlations with most of the measured parameters.

Overall, the correlation analysis revealed statistical associations among photosynthetic pigment variation, membrane lipid peroxidation, osmotic adjustment, and the measured antioxidant enzyme responses in *I. polycarpa* seedlings under simulated acidification stress. These associations indicate that multiple physiological processes varied coordinately during the treatment period. However, because correlation analysis does not establish causality, these relationships should be interpreted as coordinated physiological responses rather than evidence of direct mechanistic links among the measured variables.

## 4 Discussion

### 4.1 Physiological response patterns and a potential tolerance boundary under simulated acidification stress

The present study showed that the physiological responses of *I. polycarpa* seedlings to simulated acidification stress were strongly dependent on both acidity intensity and exposure duration. Under mild to moderate acidification, particularly at pH 5.5 and pH 4.5, seedlings showed partial physiological adjustment, as reflected by comparatively lower visible injury indices and less pronounced changes in several measured physiological parameters than those observed under pH 3.5. In contrast, the pH 3.5 treatment induced progressive visible injury, elevated MDA at specific middle and late sampling times, pronounced temporal fluctuations in photosynthetic pigments, and marked changes in the measured antioxidant enzyme activities. These results suggest that *I. polycarpa* seedlings possess a degree of physiological plasticity under simulated acidification stress, but this regulatory capacity becomes insufficient when acidity reaches a severe level. Similar coordinated changes in photosynthetic performance, cellular integrity, and stress-related physiology have also been reported in plants exposed to simulated acid rain (Ansari and Khan, 2025).

The endpoint substrate pH measurements provide descriptive information on the root-zone chemical condition at the end of the 50-d treatment period. After 50 d, the endpoint substrate pH was 6.86 in CK and was progressively lower in the acidification treatments, with values of 5.63, 5.33, and 4.99 under the pH 5.5, pH 4.5, and pH 3.5 irrigation treatments, respectively. Importantly, the endpoint substrate pH values in the acidification treatments remained higher than the nominal pH values of the applied solutions, which is consistent with a buffering effect of the peat–vermiculite substrate. However, because only one composite substrate sample was analyzed per treatment and pH was measured only at the end of the experiment, these values should not be interpreted as representing temporal changes in substrate pH or the dynamic rhizosphere conditions experienced by seedlings throughout the 50-d treatment period. Accordingly, the treatment labels used in this study represent the pH of the applied irrigation solutions rather than the actual substrate pH.

At pH 4.5, seedlings exhibited visible and biochemical stress responses, but the magnitude of physiological disturbance was generally less pronounced than that observed under pH 3.5. In contrast, the pH 3.5 treatment was associated with substantially greater cumulative visible injury, pronounced temporal fluctuations in photosynthetic pigments, and marked increases in MDA at specific later sampling times. These observations suggest that the transition from partial physiological adjustment to pronounced stress injury may occur within the tested interval between pH 4.5 and pH 3.5. However, because only three acidification levels were evaluated and no breakpoint or dose–response analysis was performed, the present data do not support definition of an exact biological threshold. Accordingly, the pH 4.5–3.5 interval is interpreted here as a potential physiological tolerance boundary under the controlled acidification conditions of this experiment. Similar stress transitions have been reported in plants exposed to acidic pH environments, where growth inhibition and physiological imbalance became more evident as acidity increased (Wang et al., 2022). Excessive acidity may disturb nutrient uptake and root function, and in natural acidic soils, acidification may also enhance Al availability and thereby contribute to plant injury (Shavrukov and Hirai, 2016; Rahman et al., 2018). However, because Al availability was not measured in the present experiment, Al toxicity cannot be identified as a demonstrated mechanism underlying the observed physiological responses.

It should be emphasized that the potential tolerance boundary suggested by this study was derived from a controlled pot experiment using repeated root-zone application of acidified solutions. It should therefore be regarded as an experimental physiological reference rather than a fixed threshold applicable to natural acidic soils. Field responses may differ because soil buffering capacity and nutrient availability can modify the actual acidification stress experienced by seedlings (Yu et al., 2020; Jia et al., 2023). Acidification may also enhance Al availability and alter soil microbial processes, further affecting plant responses under natural soil conditions (Rahman et al., 2018; Zhang et al., 2023b).

### 4.2 Stage-dependent photosynthetic pigment responses under simulated acidification stress

Photosynthetic pigments are essential for light absorption and energy transfer in plant photosynthesis (Nishiyama et al., 2011). Carotenoids also contribute to photoprotection by dissipating excess excitation energy and reducing photooxidative stress (Swapnil et al., 2021). In the present study, photosynthetic pigment contents in *I. polycarpa* seedlings showed marked stage- and treatment-dependent responses to simulated acidification stress. During the early treatment period, changes in pigment contents differed among treatments rather than showing a uniform increase. At 10 d, relatively high Chl a, Total Chl, and Car contents were observed in CK and the pH 3.5 treatment, whereas the pH 4.5 and pH 5.5 treatments generally showed smaller changes. These early responses may reflect transient physiological adjustment rather than a consistent compensatory increase in pigment accumulation. Similar short-term changes in pigment contents have been reported under acidic pH environments or other abiotic stresses (Wang et al., 2022; Zhang et al., 2023c).

However, the pigment response under pH 3.5 was not characterized by a continuous decline. Chl a, Total Chl, and Car showed marked reductions at 20 d, followed by partial recovery at 30 and 40 d, and then declined again at 50 d. Chl b also showed a pronounced late-stage decrease, reaching its lowest level at 50 d. This fluctuating pattern indicates that severe acidification induced an unstable pigment response rather than a simple progressive inhibition of photosynthetic pigment accumulation. Acidification stress may affect photosynthetic pigment accumulation by disturbing nutrient availability and plant physiological stability (Rahman et al., 2018; Zhang et al., 2023b). Because Mg is a central component of the chlorophyll molecule, acidification-induced changes in Mg availability may potentially affect chlorophyll synthesis or maintenance (Ahmed et al., 2023). However, nutrient availability was not measured in the present study, and this mechanism therefore remains hypothetical. In addition, oxidative stress under severe acidity may accelerate pigment degradation and impair photosynthetic stability (Debnath et al., 2021).

The marked reductions in carotenoid content under pH 3.5 at specific sampling times, particularly at 20 and 50 d, may indicate temporary impairment of non-enzymatic photoprotective capacity under severe acidification. Carotenoids are important non-enzymatic antioxidants in photosynthetic tissues and can protect chloroplasts by quenching reactive oxygen species and dissipating excess excitation energy (Swapnil et al., 2021). Therefore, reductions in Car content may decrease the capacity of seedlings to buffer photooxidative stress. However, because Car content partially recovered during the intermediate stages, the response should be interpreted as dynamic rather than as a sustained loss of photoprotective capacity.

In contrast, the pH 5.5 and pH 4.5 treatments generally showed smaller temporal fluctuations in photosynthetic pigment contents than the pH 3.5 treatment. This pattern is consistent with comparatively less severe physiological disturbance and suggests that partial physiological adjustment remained possible under the milder acidification treatments. Overall, the stage-dependent pigment responses, together with the greater instability observed under pH 3.5, provide additional physiological evidence supporting a potential tolerance boundary between the pH 4.5 and pH 3.5 treatments under the present experimental conditions.

### 4.3 Membrane lipid peroxidation and osmotic adjustment reveal stage-specific stress regulation

Membrane stability is highly sensitive to environmental stress, and MDA is widely used as an indicator of membrane lipid peroxidation and oxidative injury in plants (Debnath et al., 2021; Zhang et al., 2023c). In the present study, MDA content in *I. polycarpa* seedlings showed a non-monotonic response to simulated acidification stress. Under pH 3.5, MDA content was significantly elevated at several middle and late sampling times, particularly at 20, 40, and 50 d, with the highest value occurring at 40 d. This pattern indicates that severe acidification was associated with enhanced membrane lipid peroxidation at specific stages rather than a continuous increase throughout the treatment period. The elevated MDA levels at these sampling times were also consistent with the greater visible injury observed under pH 3.5.

Soluble sugars and proline are important osmotic adjustment substances involved in maintaining cellular osmotic balance and stress responses under adverse environmental conditions (Saddhe et al., 2021; Hayat et al., 2012). In the present study, however, neither soluble sugar nor proline showed a simple progressive accumulation pattern with increasing acidification duration. Soluble sugar content varied among sampling times, with relatively similar levels among treatments at 30 d, followed by a pronounced increase under pH 3.5 at 40 d and a subsequent decline at 50 d. Proline showed an even more treatment- and stage-dependent response, with the highest level observed under pH 5.5 at 30 d and under pH 3.5 at 40 d. These temporal patterns suggest that osmotic adjustment responses were dynamic and depended on both acidification intensity and exposure duration. The positive correlations of MDA with soluble sugar and proline further indicate statistical covariation among these stress-related parameters, but do not demonstrate a direct causal relationship between membrane lipid peroxidation and osmotic adjustment.

However, the osmotic adjustment response did not fully prevent physiological deterioration under prolonged severe acidification. Although increases in soluble sugar and proline were observed at specific sampling times under pH 3.5, these changes were not sustained throughout the treatment period and did not prevent the progression of visible injury. MDA content also fluctuated rather than increasing continuously. Therefore, the observed changes in osmotic adjustment substances should be interpreted as stage-specific stress responses rather than as evidence of sustained or sufficient osmotic protection under severe acidification.

Soluble protein content showed a temporal pattern distinct from those of soluble sugar and proline. It increased markedly during the early stage in all treatments, declined to relatively low levels around 30 d, partially recovered at 40 d, and then showed treatment-dependent differences at 50 d. Notably, the pH 3.5 treatment maintained a comparatively high soluble protein content at the final sampling time. Therefore, the soluble protein response cannot be interpreted as a simple progressive decline under severe acidification. Instead, it likely reflects dynamic changes in stress-related metabolism and protein turnover during different stages of the treatment.

Overall, the changes in MDA, soluble sugar, proline, and soluble protein indicate a stage- and treatment-dependent physiological response of *I. polycarpa* seedlings to simulated acidification stress. Osmotic adjustment substances showed dynamic rather than continuously increasing patterns, while MDA under pH 3.5 was elevated at specific middle and late sampling times. The pH 3.5 treatment was associated with substantially greater visible injury and more pronounced physiological disturbance than the pH 4.5 and pH 5.5 treatments. Together with the responses of the other measured physiological parameters, these patterns provide additional evidence for a potential tolerance boundary between the pH 4.5 and pH 3.5 treatments under the present experimental conditions.

### 4.4 Dynamics of the measured antioxidant enzymes under simulated acidification stress

Antioxidant enzymes are important components of plant responses to oxidative stress under adverse environmental conditions (Debnath et al., 2021; Mishra et al., 2023). In the present study, the two measured antioxidant enzymes, POD and SOD, exhibited distinct temporal and treatment-dependent responses to simulated acidification stress. POD activity increased rapidly during the early stage, particularly under pH 3.5, whereas SOD activity showed pronounced fluctuations among treatments during the middle and later stages. These patterns indicate that POD and SOD responded differently to acidification intensity and exposure duration, but they should not be interpreted as representing the complete antioxidant system of *I. polycarpa* seedlings.

The early increase in POD activity may reflect a rapid enzymatic response to oxidative pressure induced by acidification, because POD is closely involved in H_2_O_2_ scavenging and stress-related redox regulation in plants (Zhang et al., 2015; Mishra et al., 2023). In this study, the sharp increase in POD activity under pH 3.5 during the early treatment period was consistent with the transient activation of defense-related physiological responses. However, POD activity under pH 3.5 declined during prolonged treatment, whereas MDA remained elevated at several middle and late sampling times and visible injury continued to increase. This pattern indicates that the pronounced early POD response was not maintained during prolonged severe acidification.

SOD is generally regarded as a primary antioxidant enzyme that catalyzes the dismutation of superoxide radicals into H_2_O_2_, which can subsequently be scavenged by POD and other antioxidant enzymes (Debnath et al., 2021; Mishra et al., 2023). The relatively high SOD activity observed at several sampling times under pH 5.5 and pH 4.5 indicates a dynamic enzymatic response to acidification. However, because ROS accumulation and the activities of other major antioxidant enzymes were not measured, the functional consequences of these SOD changes cannot be determined directly.

It should be noted that SOD and POD represent only part of the plant antioxidant network. Other important enzymatic components, including catalase (CAT), ascorbate peroxidase (APX), and glutathione reductase (GR), as well as non-enzymatic antioxidants such as ascorbate and glutathione, were not measured in the present study. In addition, ROS accumulation was not directly quantified. Therefore, the present results provide information only on the responses of the two measured antioxidant enzymes and do not constitute a comprehensive assessment of antioxidant capacity or cellular redox status. Future studies should incorporate a broader set of enzymatic and non-enzymatic antioxidant indicators together with direct measurements of ROS accumulation.

The correlation analysis further revealed coordinated variation among the measured antioxidant enzyme activities, membrane lipid peroxidation, photosynthetic pigments, and osmotic adjustment substances. POD activity was negatively correlated with MDA, soluble sugar, and proline contents, while MDA was positively correlated with soluble sugar and proline contents. These correlations indicate statistical covariation among the measured physiological parameters but do not establish direct mechanistic relationships. However, because correlation analysis does not establish causality, these relationships should be interpreted as evidence of coordinated physiological changes rather than direct causal links.

Taken together, the results indicate a stage-specific physiological response pattern in *I. polycarpa* seedlings under simulated acidification stress. During the early stage, transient changes in photosynthetic pigments and a rapid increase in POD activity were observed. During the middle stage, changes in osmotic adjustment substances and SOD activity became more evident. Under mild to moderate acidification, these responses were accompanied by comparatively less severe physiological disturbance. In contrast, under pH 3.5, membrane lipid peroxidation was elevated at specific middle and late sampling times, photosynthetic pigments showed pronounced fluctuations with marked reductions at particular stages, the two measured antioxidant enzymes varied markedly, and visible injury increased progressively. These findings indicate that the physiological response to severe acidification was more pronounced and less stable than that observed under the milder treatments.

Overall, *I. polycarpa* seedlings showed comparatively less severe physiological disturbance under the pH 5.5 and pH 4.5 treatments than under pH 3.5. The distinct responses of POD and SOD, together with changes in MDA, photosynthetic pigments, and visible injury, further demonstrate that acidification intensity and exposure duration strongly influenced the physiological responses of the seedlings. These results are consistent with the interpretation of a potential physiological tolerance boundary between the pH 4.5 and pH 3.5 treatments under the present experimental conditions. However, because only two antioxidant enzymes were measured and no direct ROS measurements were available, the contribution of the overall antioxidant system to acidification tolerance cannot be fully determined from the present dataset.

### 4.5 Implications, limitations, and future validation

Although this study was conducted under controlled pot conditions using root-zone irrigation with acidified solutions, the results provide preliminary physiological evidence for evaluating the acidification tolerance of *I. polycarpa* seedlings. The comparatively less severe visible injury and physiological disturbance observed under pH 5.5 and pH 4.5 suggest that *I. polycarpa* seedlings exhibited partial physiological adjustment under mild to moderate acidification. In contrast, the pronounced visible injury, marked temporal fluctuations in photosynthetic pigments, elevated MDA at specific middle and late sampling times, and pronounced changes in the two measured antioxidant enzymes under pH 3.5 indicate substantially greater physiological disturbance under severe acidification. Therefore, the potential application of *I. polycarpa* in acidic or acidification-prone sites should be approached cautiously, especially where rhizosphere acidity may reach strongly acidic levels.

The chemical environment generated by the acidification treatments also requires cautious interpretation. Although endpoint substrate pH was measured, only one composite substrate sample was available for each treatment, and therefore these measurements were descriptive rather than suitable for inferential statistical comparison. In addition, substrate buffering capacity, electrical conductivity, sulfate accumulation, changes in nutrient availability during and after the acidification treatment, and exchangeable or soluble Al were not quantified. Accordingly, Al toxicity is discussed only as a possible mechanism reported for natural acidic soils and not as a process demonstrated in the present experiment. Consequently, the physiological responses observed in this study cannot be attributed specifically to proton toxicity, sulfate accumulation, nutrient imbalance, Al mobilization, or any single chemical component of acidification. Accordingly, interpretations involving ROS accumulation, broader antioxidant regulation, Al mobilization or toxicity, and changes in substrate chemistry should be regarded as possible mechanisms or directions for future investigation rather than processes directly demonstrated by the present dataset. In addition, all treatment solutions were prepared with deionized water, which differs in ionic composition from natural precipitation and soil water and may have influenced nutrient dynamics during the 50-d treatment period. The present experiment should therefore be interpreted as a controlled assessment of seedling responses to repeated root-zone application of acidified solutions rather than as a complete simulation of natural acidic-soil processes. Future studies should combine finer acidity gradients with repeated measurements of substrate chemistry to distinguish the contributions of these potential mechanisms.

The cumulative injury index should also be interpreted as a descriptive cohort-level indicator rather than a contemporaneous assessment of all 15 seedlings at each sampling time. Because three seedlings per treatment were destructively harvested at each post-treatment sampling time, the last recorded injury grade of previously harvested seedlings was retained in subsequent cumulative calculations. Therefore, the cumulative injury index reflects the progression of injury within the original treatment cohort but does not represent repeated direct observation of all individuals throughout the full 50-d period.

A further limitation is that antioxidant responses were evaluated only through SOD and POD activities. Because CAT, APX, GR, non-enzymatic antioxidants, and ROS accumulation were not measured, the overall antioxidant capacity and redox status of the seedlings cannot be fully evaluated from the present dataset.

Another limitation is the relatively small number of biological replicates used for physiological and biochemical measurements (n = 3 per treatment at each sampling time). Although each replicate represented an independent seedling and different seedlings were destructively sampled at successive time points, the limited replication may reduce statistical power, particularly for detecting small or moderate treatment effects. Therefore, the statistical differences and temporal response patterns observed in this study should be interpreted cautiously. Future studies with larger numbers of independent biological replicates will be necessary to improve statistical power and verify the reproducibility of the observed responses.

Despite these limitations, this study helps identify key physiological indicators that may be useful for screening acidification tolerance in *I. polycarpa* seedlings, including photosynthetic pigment stability, MDA accumulation, osmotic adjustment substances, and antioxidant enzyme dynamics. These indicators may support future evaluation of woody oil tree species for cultivation in acidic or acidification-prone environments.

## 5 Conclusion

This study characterized the dynamic physiological responses of one-year-old *I. polycarpa* seedlings to controlled root-zone acidification. The responses were strongly dependent on both acidification intensity and exposure duration. These responses should be interpreted as treatment-dependent physiological adjustments under the present controlled experimental conditions. Under the pH 5.5 and pH 4.5 irrigation treatments, seedlings showed comparatively less severe visible and physiological disturbance, whereas the pH 3.5 treatment was associated with substantially greater cumulative injury, pronounced temporal fluctuations in photosynthetic pigments and osmotic adjustment substances, elevated MDA at specific middle and late sampling times, and marked changes in the two measured antioxidant enzymes. These findings suggest that a potential physiological tolerance boundary may occur between the pH 4.5 and pH 3.5 irrigation treatments under the present controlled conditions. However, because only three acidification levels were tested and no breakpoint or dose–response modeling was performed, this boundary should be regarded as a preliminary physiological reference rather than an exact biological threshold. Further studies using finer acidity gradients, larger biological replication, more comprehensive characterization of substrate chemistry and antioxidant responses, and natural acidic soils are needed to validate and refine this potential response boundary.

## Data Availability

The raw data supporting the conclusions of this article will be made available by the authors, without undue reservation.
